# Development and validation of a prognostic prediction model including the minor lymphatic pathway for distant metastases in cervical cancer patients

**DOI:** 10.1038/s41598-022-13616-0

**Published:** 2022-06-14

**Authors:** Kullathorn Thephamongkhol, Pornpim Korpraphong, Kobkun Muangsomboon, Chomporn Sitathanee, Arb-aroon Lertkhachonsuk, Sith Phongkitkarun, Saowanee Srirattanapong, Duangkamon Prapruttam, Jidapa Bridhikitti, Thaworn Dendumrongsup, Petch Alisanant, Napapat Amornwichet, Chonlakiet Khorprasert, Kewalee Sasiwimonphan, Chamnan Tanprasertkul, Mantana Dhanachai, Jayanton Patumanond, Jiraporn Setakornnukul

**Affiliations:** 1 Division of Radiation Oncology, Department of Radiology, Faculty of Medicine, Siriraj Hospital, Mahidol University, 2 Wanglang Road, Bangkok Noi, Bangkok, 10700 Thailand; 2 Division of Diagnostic Radiology, Department of Radiology, Faculty of Medicine, Siriraj Hospital, Mahidol University, Bangkok, Thailand; 3 Division of Radiation and Oncology, Department of Diagnostic and Therapeutic Radiology, Faculty of Medicine, Ramathibodi Hospital, Mahidol University, Bangkok, Thailand; 4 Gynecological Oncology Unit, Department of Obstetrics and Gynecology, Faculty of Medicine, Ramathibodi Hospital, Mahidol University, Bangkok, Thailand; 5 Division of Diagnostic Radiology, Department of Diagnostic and Therapeutic Radiology, Faculty of Medicine, Ramathibodi Hospital, Mahidol University, Bangkok, Thailand; 6 Division of Radiation Oncology, Department of Radiology, Faculty of Medicine, Songklanagarind Hospital, Prince of Songkla University, Hat Yai, Songkhla Thailand; 7 Abdominal Imaging Section, Department of Radiology, Faculty of Medicine, Songklanagarind Hospital, Prince of Songkla University, Hat Yai, Songkhla Thailand; 8 Division of Therapeutic Radiology and Oncology, Department of Radiology, King Chulalongkorn Memorial Hospital, Faculty of Medicine, Chulalongkorn University, Bangkok, Thailand; 9 Division of Diagnostic Radiology, Department of Radiology, King Chulalongkorn Memorial Hospital, Faculty of Medicine, Chulalongkorn University, Bangkok, Thailand; 10 Minimally Invasive Gynecologic Unit, Department of Obstetrics and Gynecology, Faculty of Medicine, Thammasat University, Pathum Thani, Thailand; 11 Department of Clinical Epidemiology, Faculty of Medicine, Thammasat University, Pathum Thani, Thailand; 12 Center for Clinical Epidemiology and Clinical Statistics, Faculty of Medicine, Chiang Mai University, Chiang Mai, Thailand

**Keywords:** Cancer imaging, Cervical cancer

## Abstract

To develop and validate a prognostic model, including the minor lymphatic pathway (internal iliac and presacral nodes). Study design: Retrospective cohort. Participants: Locally advanced cervical cancer underwent concurrent chemoradiotherapy. Sample size: 397 and 384 patients in the development and validation data set. Predictors: Our new nodal staging system with the minor lymphatic pathway. Outcome: Distant metastases. Statistical analysis: Cox regression; net reclassification improvement (NRI) and decision curve analysis (DCA). Our new nodal system was the strongest predictor. The predictors in the final model were new nodal system, tumor stage, adenocarcinoma, initial hemoglobin, tumor size and age. The nodal system and the pretreatment model had concordance indices of 0.661 and 0.708, respectively, with good calibration curves. Compared to the OUTBACK eligibility criteria, the nodal system showed NRI for both cases (22%) and controls (16%). The pretreatment model showed NRI for cases (31%) and controls (18%). DCA in both models showed threshold probability of 15% and 12%, respectively, when compared with 24% in OUTBACK eligibility criteria. Our new nodal staging system and the pretreatment model could differentiate between high-risk and low-risk patients, thus facilitating decisions to provide more aggressive treatment to prevent distant metastases.

## Introduction

Distant metastases are now the leading cause of death in advanced cervical cancer^1^. This type of failure is increasingly common due to the improved local control now available with advanced radiotherapy^2^.

To prevent distant metastases, many large clinical trials of adjuvant chemotherapy have been investigated. There has been concern that the therapeutic ratio is very narrow. In one trial, although there were slightly fewer distant metastases in the adjuvant chemotherapy arm in Adjuvant Chemotherapy in Locally Advanced Cervical Cancer Patients (ACTLACC) trial^3^, this benefit did not translate into an overall survival benefit. Additionally, two large clinical trials showed very high toxicity^4,5^ and toxic death^5^. Consequently, adjuvant chemotherapy is still not standard practice. This could be due to either ineffective treatment effect and unacceptable toxicity or inadequate identification of high-risk patients for distant metastases. Furthermore, in the present era of personalized medicine, there is more focus on individual results, not on average results^6^. As a result, the optimum solution is to select patients with a high risk of distant metastases for chemotherapy, thus avoiding unnecessary exposure of other patients to this aggressive therapy.

The excellent and well-known model of the Korean Gynecologic Oncology Group^7^ can be used to correctly identify patients with a high risk of distant metastases. The model has been shown to have very good discrimination performance, with optimism-corrected concordance indices of 0.70 and 0.73 for development and validation data sets, respectively. The model has four parameters: pelvic and para-aortic node positivity in FDG-PET (Fluorodeoxyglucose positron emission tomography), nonsquamous cell histology, and serum levels of squamous cell carcinoma antigen before treatment. However, the use of the model is limited in developing countries due to the high costs of FDG-PET.

Given the unsolved distant metastasis problem and the limited use of the excellent Korean prediction model, we hypothesized that a solution could be the use of computed tomography with a comprehensive reading of all aspects of the lymphatic pathway^8–10^. This approach capitalizes on the widespread availability of computed tomography around the world while avoiding the need for relatively expensive PET scanners. In previous work, our team found that minor lymphatic pathways with two or more lymph node metastases were highly associated with distant metastases^11^. This association might be explained by a direct connection between the lymphatic and venous circulations in the pelvis^12–15^. We integrated this new prognostic factor with the criteria from the EMBRACE study (Image guided intensity modulated External beam radiochemotherapy and Magnetic-resonance-imaging based adaptive BRAchytherapy in locally advanced CErvical cancer)^16^. Under the EMBRACE criteria, high-risk cases are tested to determine their suitability for adjuvant chemotherapy. We termed the resulting integration our new nodal staging system. Subsequently, several distant metastasis predictors were added to form a full clinical prediction model.

In this study, the nodal staging system and full models were tested to determine whether they are suitable for use as new eligibility criteria in clinical trials. Furthermore, they were evaluated to determine their value in making individual predictions of the risk of distant metastases to support clinical judgments on the need for aggressive systemic treatment.

## Methods

We used retrospective cohort data from four tertiary care hospitals in Thailand. For the development set, we used data for 2007–2012 from one university hospital (Siriraj Hospital). As to the validation set, we used data for 2007–2015 from three university hospital (Ramathibodi Hospital, King Chulalongkorn Memorial Hospital and Songklanagarind Hospital). Follow-up data were collected from all four hospitals until May 2018.

Eligible participants were women aged 18 years and older with locally advanced cervical cancer. The patients were routinely treated with definitive chemoradiotherapy with curative intent. For staging purposes, all patients underwent a CT scan with contrast media of the entire abdomen, chest X-ray examination, and cystoscopy. We excluded patients who had inadequate chemoradiotherapy due to a poor performance status or who had preexisting distant metastases.

Our main predictors of lymph nodes were investigated in our previous study, in which the lymphatic pathways were comprehensively reviewed. We found the minor lymphatic pathway was highly associated with distant metastases. Based on this result and high risk group of EMBRACE system, we proposed the new nodal staging system. It had 4 risk categories: low risk (No lymph node metastases; N0); intermediate risk (not low risk; no high-risk features; N1); high risk (≥ 1 pathological node in the common iliac or above, or ≥ 3 pathological nodes in pelvic node; N2); and very high risk (≥ 2 presacral or internal iliac nodes; N3). Other predictors were known clinical predictors: clinical T stage (according to the FIGO 2009 staging system); initial hemoglobin (g/dL); histology (squamous cell carcinoma, adenocarcinoma, and adenosquamous carcinoma); tumor size (centimeters); and patient age (years) at diagnosis. We also used two posttreatment predictors: a treatment time of more than 55 days (yes or no), and the treatment response one month after radiation completion (response or not). As all clinical predictors were collected by research assistants, the process was blinded to lymph node predictors and outcomes. Also, evaluations of the lymph node predictors were performed by diagnostic radiologists and outcomes were recorded by radiation oncologists and gynecological oncologists. Therefore, this process was also blinded from the evaluations of the other predictors and outcomes.

The main outcome of this study was a first failure in the form of visceral distant metastases or lymph node metastases above the diaphragm. We excluded secondary metastases following the first loco-regional recurrence. Generally, supraclavicular lymph node metastases were diagnosed by pathology, whereas multiple lung, liver or bone metastases were diagnosed by imaging. Our routine follow-up consisted of a physical and pelvic examination. Imaging was performed at the discretion of the treating physician.

### Sample size

Regarding the calculation of the study size, we used 10 events per variable as per the TRIPOD guidelines^17^. If we expected 20% of distant metastases from about 400 patients in the development and validation dataset each needed at least 80 events and at least 400 patients.

### Statistical analysis

Comparisons of the patient characteristics of the development and validation data sets were made. An unadjusted analysis of each variable was also performed using Cox regression.

For the model building, we handled the predictors as per the TRIPOD guidelines^17^. To develop the models, we performed Cox regression using variable selection with the backward elimination approach of each subset of variables, and an Akaike criterion *P* value of 0.157. We then decided to force variables recognized as clinical importance back into the model. To assess and correction of optimism, we performed internal validation of each using the bootstrapping method, resampling 1000 times with replacement for optimism correction using the validate function in the RMS package (version 6.2-0 by Professor Harrell)^18^ in R software version 4.05 (https://www.r-project.org)^19^. We hypothesize that our new nodal staging system could be the simplified model. It was tested using the Breiman permutation method^20^ for computing the relative importance of new nodal staging system in a survival model^21^.

We used standard-model performance measures for discrimination and calibration. Discrimination was measured with Harrell’s concordance index and the 95% confidence interval. For the calibrations of the development and validation data sets, we used 2 calibration curve methods. The first method used population-averaged survival curve based on 3 risk groups developed by Professor Royston (< 25 percentile, 25–75 percentile, and > 75 percentile of linear predictor). By using the STCOXGRP command^22^ in STATA software version 17.0 (https://www.stata.com)^23^, the whole lines of the mean predicted survival probabilities were plotted from the smoothed baseline log cumulative hazard function versus the 95% confidence intervals of the observed probabilities. The second calibration curve method, calibration was assessed by the plot of each deciles of the 5-year predicted probabilities versus the 5-year observed probabilities. For this purpose, the PMCALPLOT module (revised Jan 4, 2020)^24^, of STATA software^23^ was used.

For specification of our two full models (pretreatment and posttreatment models), we used the coefficient and baseline survival at 60 months from Cox regression to calculate 5-year risk of distant metastases. We used the following groups: low-risk (< 15% risk of distant metastases); intermediate-risk (15% to < 30% risk of distant metastases); and high-risk (≥ 30% risk of distant metastases). On the other hand, we used the original categorization for the new nodal staging system, OUTBACK eligibility criteria, ACTLACC eligibility criteria and EMBRACE criteria.

To quantify how precisely new model can separate high risk from low risk patients in clinical aspect, net reclassification improvement (NRI) was used. NRI measured how many distant metastasis cases were corrected to higher risk groups (NRI+), and how many controls (no distant metastases) were corrected to lower risk groups (NRI-). The NRI values were then summation of NRI+ and NRI−, using NRICENS package (version 1.6)^25^ in R software (version 4.05)^19^. To have more clinical sense, we also use specific subgroups of standard eligibility criteria in OUTBACK and high risk group of original EMBRACE with proportionally reported 100 patients of each subgroup for calculating NRI. These patient populations were supposed to be debated for adjuvant chemotherapy.

A decision curve analysis using the STDCA command in the STATA software were done. The method accounts for censored observations and was developed by Professor Vickers of the Memorial Sloan Kettering Cancer Center^26^. The main concept is to compare the benefits of models when varying the probability threshold (the probability of deciding to have treatment or not). If a new model produces a net benefit at a lower probability threshold than another model, the new model is more useful for patients and physicians.

In the case of the external validation, we used the previous coefficient and baseline survival at 60 months obtained from the development model for testing with the validation data set. However, for validation of the models with original categorizations (OUTBACK, ACTLACC, original EMBRACE and our new nodal staging system), we used their standard categorizations rather than the coefficient for the validation data set. We used two additional quantitative methods of calibration recommended by Professor Royston and Professor Altman^27^. In the first of those methods, a comparison was made of the differences in the hazard ratios of the risk groups in the development data set and the validation data set. The second method involved putting the linear predictor in the validation data set, followed by all of the other variables. The *P* values of all variables except the linear predictor were calculated. Any variable with a *P* value > 0.05 was regarded as a misspecification.

### Institutional review board statement

The study was conducted according to the guidelines of the Declaration of Helsinki, and approved by the Institutional Review Board of Faculty of Medicine, Siriraj Hospital, Mahidol University (Si 312/2016, 12 May 2016).

### Informed consent

Patient consent was waived due to Institutional Review Board (IRB) policy of retrospective chart review study. This was allowed by the Institutional Review Board of Faculty of Medicine, Siriraj Hospital, Mahidol University (Si 312/2016, 12 May 2016).

## Results

### Participant flow

In all, 432 eligible cases with computed tomography scans were available for the development dataset. After excluding 35 patients without curative intent treatments (23 without chemotherapy, and 12 without brachytherapy), 397 patients were considered for inclusion in the development data set (Supplementary Fig. S1).

### Participant characteristics

A total of 397 patients were included in development data set (Table 1). Majority of the patients (83%) were diagnosed with FIGO (2018) stage IIIB or higher. Around half of the patients had lymph node metastases, of these 31% at pelvic area only (stage IIIc1), 18% at para-aortic area (stage IIIc2) (Table 1). In patients with para-aortic lymph nodes, the majority (80%) of these had positive lymph nodes below left renal vein, which were classified as level 326 b1 according to Japan Society of Gynecologic Oncology (Supplementary Table S1).

**Table 1:** Comparison of baseline of patients and treatment characteristics with the number of distant metastases and unadjusted analysis in the development data set.

Characteristics	All patients (N = 397)	Distant metastases N (%)		HR (95% CI)	*P* value*
		Yes = 93	No = 304		
**Demographic and tumor**					
Age, mean ± SD	55.1 (11.8)	56.5 (12.0)	54.6 (11.7)	1.01 (0.99–1.03)	0.219
Initial hemoglobin (g/dl)	11.4 (1.8)	11.1 (1.9)	11.6 (1.8)	0.84 (0.75–0.94)	0.002
Histology					0.149
SCC + AdenoSCC	327 (82.4)	72 (77.4)	255 (83.9)	1 (Reference)	
AdenoCA	70 (17.6)	21 (22.6)	49 (16.1)	1.49 (0.91–2.42)	
Tumor size, mean ± SD	4.4 (1.5)	4.6 (1.5)	4.4 (1.5)	1.16 (1.01–1.32)	0.030
**Staging**					
FIGO 2018					< 0.001
I–II	67 (16.9)	9 (9.7)	58 (19.1)	1 (Reference)	
IIIB	132 (33.3)	23 (24.7)	109 (35.9)	1.43 (0.66–3.08)	
IIIIC1	124 (31.2)	33 (35.5)	91 (29.9)	2.51 (1.20–5.24)	
IIIC2	70 (17.6)	26 (28.0)	44 (14.5)	3.98 (1.86–8.50)	
IVA	4 (1.0)	2 (2.2)	2 (0.7)	4.39 (0.95–20.36)	
T stage only					0.001
IB1-IIB	200 (50.4)	35 (37.6)	165 (54.3)	1 (Reference)	
IIIA-IVA	197 (49.6)	58 (62.4)	139 (45.7)	2.03 (1.34–3.09)	
OUTBACK					0.001
Less than EC**	5 (1.3)	2 (2.2)	3 (1.0)	1 (Reference)	
EC	322 (81.1)	65 (69.9)	257 (84.5)	0.59 (0.15–2.43)	
More than EC	70 (17.6)	26 (28.0)	44 (14.5)	1.38 (0.33–5.82)	
Original EMBRACE					< 0.001
Low risk	9 (2.3)	2 (2.2)	7 (2.3)	1 (Reference)	
Intermediate risk	212 (53.4)	32 (34.4)	180 (59.2)	0.78 (0.19–3.29)	
High risk	176 (44.3)	59 (63.4)	117 (38.5)	2.18 (0.53–8.93)	
New nodal staging system					< 0.001
Low risk (N0)	134 (33.8)	16 (17.2)	118 (38.8)	1 (Reference)	
Intermediate risk (N1)	86 (21.7)	17 (18.3)	69 (22.7)	1.78 (0.90–3.52)	
High risk (N2)	160 (40.3)	50 (53.8)	110 (36.2)	3.31 (1.88–5.81)	
Very high risk (N3)	17 (4.3)	10 (10.8)	7 (2.3)	7.86 (3.56–17.38)	
**Treatment and response**					
Treatment time					0.004
≤ 55 days	310 (78.1)	64 (68.8)	246 (80.9)	1 (Reference)	
> 55 days	87 (21.9)	29 (31.2)	58 (19.1)	1.92 (1.24–2.99)	
Response at 1 month					0.033
Response	372 (93.7)	87 (93.5)	285 (93.8)	1 (Reference)	
No response	25 (6.3)	6 (6.5)	19 (6.3)	2.48 (1.07–5.75)	

*P value of test parameters from univariable Cox regression.

**EC Eligibility criteria.

Approximately 80% and 40% of all patients were candidates for adjuvant chemotherapy according to OUTBACK eligibility criteria and high risk group of the EMBRACE criteria respectively (Table 1). Importantly, 1 patient (0.5%) in the intermediate-risk group and 16 patients (9%) in the high-risk group of the EMBRACE criteria were upstaged to the very high-risk group in our nodal staging system. Also, 125 patients (59%) from the intermediate-risk group of EMBRACE criteria were downstaged to the low-risk group of our nodal staging system.

In a comparison with the 384 patients in the validation data set (Supplementary Table S2), the development data set showed the proportion of patients with stage IIIC2 cancer was higher (development data set, 17.6%; validation data set, 8.3%). Distant metastases were slightly more common among the patients in the development data set than among those in the validation data set (23.4% vs 19.8%), as was death (44.3% vs 32.3%). Furthermore, the centralized linear predictor confirmed this comparison, showing that median was slightly higher for the development data set (Supplementary Fig. S2).

### Effect of univariable analysis

There was clearly an increased chance of distant metastases from stages I–II to stage IVA. Interestingly, the intermediate-risk group of OUTBACK and the original EMBRACE showed a lowest risk of distant metastases. However, the nodal staging system clearly showed a progressively increasing chance of distant metastases from the low-risk group through to the intermediate-, high-, and very high-risk groups (Table 1).

### Model development

By using the backward elimination approach (Supplementary Table S3), the 6 variables in the pretreatment model were new nodal staging system; clinical T stage, adenocarcinoma histology; pretreatment hemoglobin level; tumor size; and patient age. To form a posttreatment model, we added 2 variables after treatment: overall treatment time exceeding 55 days, and tumor response 1 month after treatment completion.

### Model specification

We obtained coefficients for the final model using the development data set. We used the original categorization for the new nodal staging system, OUTBACK eligibility criteria, ACTLACC eligibility criteria and EMBRACE criteria) we also provided an example of how to use the models (Table 2 and Supplementary Table S4).

**Table 2:** Model specification comparison and example of 36-year-old woman diagnosed with squamous cell carcinoma of cervix as T3B with diameter 6 cm, 2 or more minor lymphatic pathways (presacral and internal iliac node), and initial hemoglobin of 13.4 mg/dl.

Model	Risk group	Definition	Case example
OUTBACK (category)	Low risk	Stage less than eligibility criteria (stage IB1, FIGO2009)	FIGO 2009 IIIB with positive node = eligibility criteria = intermediate risk
	Intermediate risk	Eligibility of trial (stage IB1N + to IVA, FIGO2009)	
	High risk	Stage more than eligibility criteria (positive para-aortic node)	
Original EMBRACE (category)	Low risk	Tumor size ≤ 4 cm and stage IA, IB1, IIA1 (FIGO 2009) and N0 and squamous cell CA and No uterine invasion	Tumor ≥ 4 cm, 2 node positive but in pelvis = intermediate risk
	Intermediate risk	Not low risk, no high-risk features	
	High risk	≥ 1 pathological node at common iliac or above OR ≥ 3 pathological nodes	
Our new nodal staging system (category)	Low risk (N0)	Negative node	Positive 2 or more minor lymphatic pathway = high risk
	Intermediate risk (N1)	Not low risk, no high-risk features	
	High risk (N2)	≥ 1 pathological node at common iliac or above OR ≥ 3 pathological nodes	
	Very high risk (N3)	≥ 2 internal iliac nodes or presacral nodes (minor lymphatic pathway)	
Full model before treatment (probability prediction then grouping by 15%, 30% risk)	Low risk	< 15% of distant metastasis	Linear predictor (LP) = .5325992*0 + 1.072855*0 + 1.836307*1 + .492125*1 + .7604686*0 + − .1128717*(13.4–11.44861461) + .081321* (6–4.412989926) + .013441*(36–55.07808564) Linear predictor = 1.9808045 5-year risk of distant metastasis = 1 − (0.9010479^ exp (1.9808045*0.8713)) = 43.31% = high risk group
	Intermediate risk	15–30% of distant metastasis	
	High risk	> 30% of distant metastasis	
	Baseline survival at 60 months (S0_60m) = 0.9010479 Shrinkage factor = 0.8713 Linear predictor (LP) = .5325992*intermediate risk + 1.072855*high risk + 1.836307*very high risk + .492125*clinicalT34 + .7604686*adeno − .1128717* (initialHb (g/dL)-11.44861461) + .081321* (tumorsize (cm)-4.412989926) + .013441*(Age (years)-55.07808564) 5-year risk of distant metastasis = 1 − (S0_60m^ exp(LP*0.8713))		

#### The most important variable: our new nodal staging system (the simplified model)

**Figure 1:**
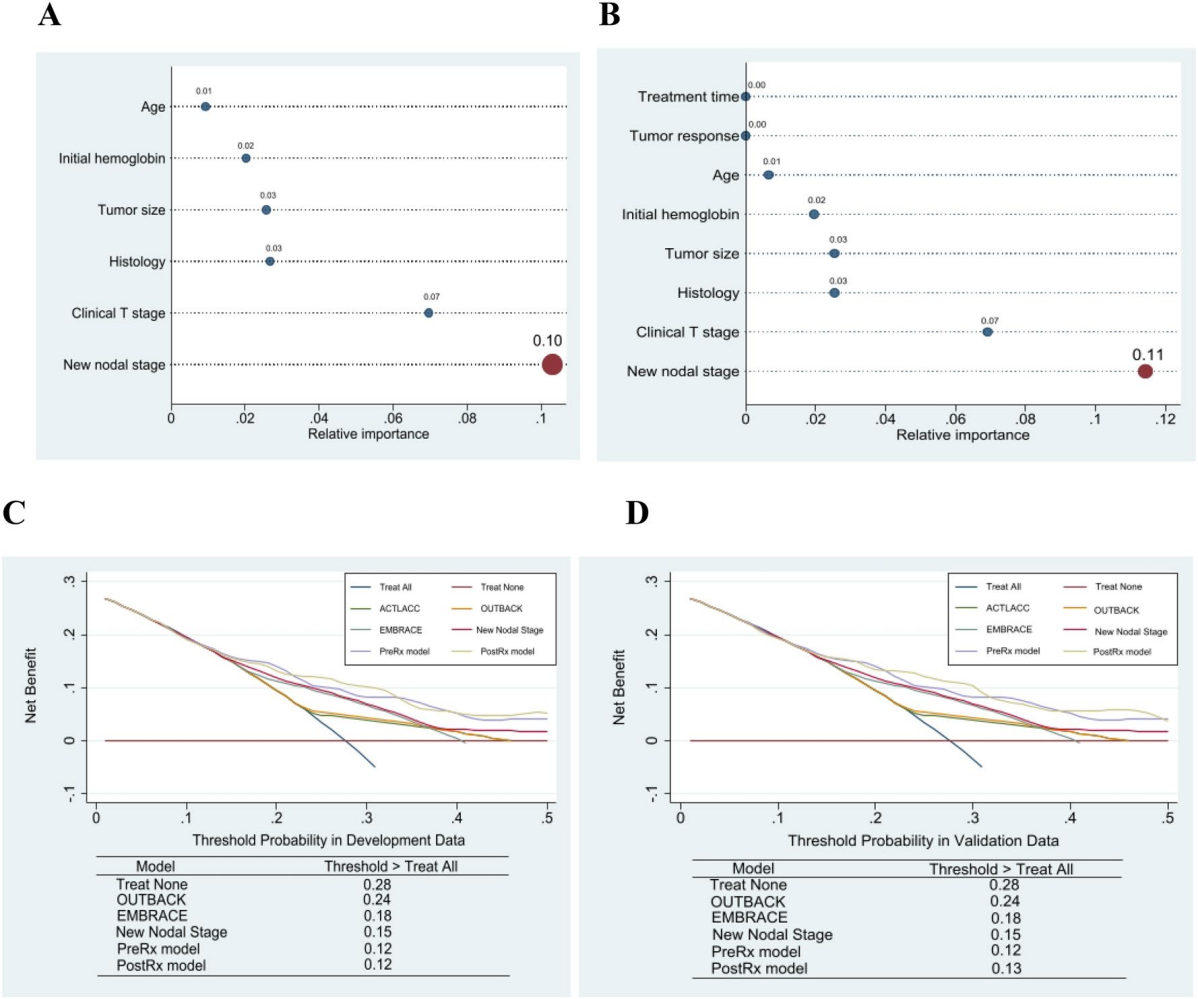
Variable importance by the Breiman permutation method in the pretreatment model (**A**) and the posttreatment model (**B**). Decision curve analysis in development dataset (**C**) and validation dataset (**D**).

We observed that the new nodal staging system was a very strong predictor in the pretreatment and the posttreatment model. The Breiman permutation method gave a relative importance of 0.10 and 0.11 in pretreatment and posttreatment models respectively (Fig. 1A,B), which confirmed the new nodal staging system was the most important variable. Therefore, we decided to use the new nodal staging system as our simplified model.

#### Model performance

**Figure 2:**
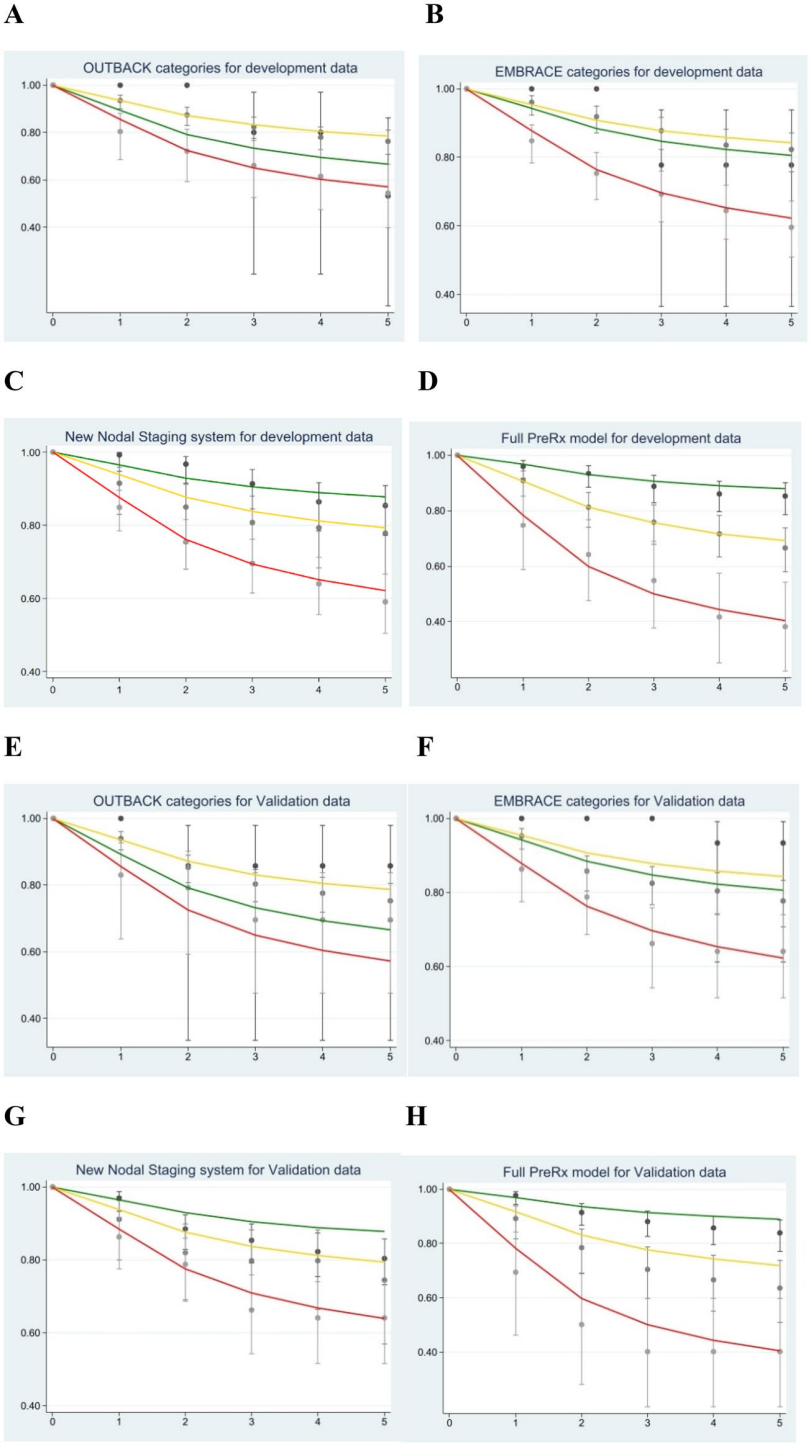
Calibration performance of development data set in OUTBACK (**A**), EMBRACE (**B**), the new nodal staging system (**C**), the pretreatment model (**D**) and of validation data set in OUTBACK (**E**), EMBRACE (**F**), the new nodal staging system (**G**), the pretreatment model (**H**).

We found that the discrimination performances of the pretreatment and posttreatment models were very similar (0.708 and 0.716 in the development data set, and 0.706 and 0.718 in the validation data set, respectively; Table 3 and Supplementary Table S5). Discrimination performance in the development data set decreased by approximately 2% after optimism correction. The new nodal staging system has a lower discrimination performance of about 4% compared with that of the pretreatment model (0.661 and 0.614 in the development and validation data sets, respectively; Table 3). Calibration of all models demonstrated a good fit with the observed data in the development data set (Fig. 2C,D,G,H and Supplementary Figs. S3, S4). As the pretreatment and posttreatment models had almost the same performances and, we mainly describe the pretreatment model. The posttreatment model is described in more detail in the supplementary files.

**Table 3:** Comparison of discrimination performance of models using development and validation data sets.

	OUTBACK	Original	Simplified model	Full model
	Eligibility criteria	EMBRACE (to level A1*)	New nodal staging system	Prognostic model before treatment
**Development**				
Number of patients	397	397	397	397
Number of events	93	93	93	93
**Variables**				
OUTBACK**				
Less than EC	1 (Reference)	–	–	–
EC	0.59 (0.15–2.43)	–	–	–
More than EC	1.38 (0.33–5.82)	–	–	–
EMBRACE*				
Low risk	–	1 (Reference)	–	–
Intermediate risk	–	0.79 (0.19–3.29)	–	–
High risk	–	2.18 (0.53–8.93)	–	–
New nodal system				
Low risk	–	–	1 (Reference)	1 (Reference)
Intermediate risk	–	–	1.78 (0.90–3.52)	1.70 (0.85–3.41)
High risk	–	–	3.31 (1.89–5.81)	2.92 (1.64–5.21)
Very high risk	–	–	7.86 (3.56–17.38)	6.27 (2.76–14.26)
T stage only				
IB1–IIB	–	–	–	1 (Reference)
IIIA–IVA	–	–	–	1.64 (1.04–2.57)
Histology				
SCC + adenoSCC	–	–	–	1 (Reference)
AdenoCA	–	–	–	2.14 (1.29–3.55)
Initial Hb	–	–	–	0.89 (0.79–1.01)
Tumor size	–	–	–	1.08 (0.93–1.26)
Age	–	–	–	1.01 (0.99–1.03)
Cox 2-yr DM*** (%)	14.48	13.90	13.42	12.50
Cox 3-yr DM (%)	20.17	19.44	18.86	17.91
Cox 5-yr DM (%)	27.05	26.13	25.51	24.77
C-statistics (95%CI)	0.574 (0.527–0.621)	0.630 (0.580–0.679)	0.661 (0.613–0.709)	0.708 (0.653–0.761)
1-Optimism	0.9311	0.9654	0.9582	0.8713
Optimism corrected	0.571 (0.524–0.618)	0.625 (0.577–0.673)	0.658 (0.610–0.706)	0.685 (0.636–0.734)
**Validation**				
Number of patients	384	384	384	384
Number of events	76	76	76	76
C-statistics (95%CI)	0.522 (0.480–0.564)	0.574 (0.514–0.635)	0.614 (0.553–0.675)	0.706 (0.653–0.760)

*EMBRACE extended to level A1 (node level just below diaphragm) in order to fairly compare with other models.

**OUTBACK: Less than eligibility criteria = IA2-IB1, eligibility criteria = IB1N + to IVA, more than eligibility criteria = PAN+.

***DM Distant metastasis rate.

Remarkably, when compared with the OUTBACK and ACTLACC eligibility criteria (Table 3 and Supplementary Table S5), our pretreatment model clearly demonstrated a better discrimination performance: up to 12 percent (0.708 vs 0.574) in the development data set, and up to 19 percent (0.706 vs 0.522) in the validation data set. In the same way, our pretreatment model (Table 2) was also 6% to 7% higher than with the EMBRACE criteria (0.708 vs 0.630 before optimism correction, and 0.685 vs 0.625 after optimism correction). The calibration curves from OUTBACK, ACTLACC and EMBRACE showed miscalibrations in the low- and intermediate-risk groups with both the development and validation data sets (Fig. 2A,B,E,F and Supplementary Fig. S3), whereas the pretreatment model only showed an underestimation of risk in the validation data set. We also found that the calibration performance of our pretreatment model was more accurate. Moreover, the hazard ratios of our pretreatment model seen in the development data set were well-maintained in the validation data set (Supplementary Table S6). We also have done sensitivity analysis by removing patients treated with monthly chemotherapy and prophylactic paraaortic radiotherapy. Generally, we found that discriminative performance and calibration curve of 4 models in development data set were about the same. On the other hand, the discriminative performance of new nodal system was increased about 6% (0.614 to 0.675). (Supplementary Table S7 and Supplementary Fig. S5).

### Net reclassification improvement (NRI) and decision curve analysis (DCA)

Clearly, we found that both our pretreatment model and our nodal staging system have more overall net reclassification improvement (NRI) than that of OUTBACK eligibility criteria and EMBRACE criteria (Supplementary Table S8). These NRIs ranged from 22 to 48% and 10% to 53% in development data set and validation data set respectively.

Interestingly, all of our models also showed more appropriate reclassification in both cases and controls (NRI+ and NRI−) when compared with OUTBACK eligibility criteria (Table 4). These clear benefits were not only up to 48% more true cases (developing distant metastases), but also up to 18% more controls (free from distant metastases).

**Table 4:** Reclassification table in subgroup of OUTBACK eligibility criteria and high risk group of EMBRACE criteria.

Simulated N= 100	Our new nodal staging system						
		Develop			Validation		
		N	DM N (%)	No DM N (%)	N	DM N (%)	No DM N (%)
OUTBACK (inclusion criteria)	N0: Lower risk	38	6 (16%) (under Rx)	32 (84%) (corrected)	67	21 (31%) (under Rx)	46 (69%) (corrected)
	N1: No change	28			16		
	N2: High risk	31	12 (39%) (corrected)	19 (61%) (over Rx)	16	12 (75%) (corrected)	4 (25%) (over Rx)
	N3: Very high risk	3	2.6 (87%) (corrected)	0.4 (13%) (over Rx)	1	1 (100%) (corrected)	0 (over Rx)
EMBRACE (high risk)	N2: No change	90			95		
	N3: Very high risk	10	7 (70%) (corrected)	3 (30%) (over Rx)	5	5 (100%) (corrected)	0 (over Rx)

Simulated N = 100	Pre-treatment model						
		Develop			Validation		
		N	DM N (%)	No DM N (%)	N	DM N (%)	No DM N (%)
OUTBACK (inclusion criteria)	Lower risk	34	3 (9%) (under Rx)	31 (91%) (corrected)	49	8 (16%) (under Rx)	41 (84%) (corrected)
	No change	37			33		
	High risk	29	15 (52%) (corrected)	14 (48%) (over Rx)	18	13 (72%) (corrected)	5 (28%) (over Rx)
EMBRACE (high risk)	Lower risk	27	6 (22%) (under Rx)	21 (78%) (corrected)	37	25 (67%) (under Rx)	12 (33%) (corrected)
	No change	73			63		

This is not the case for EMBRACE criteria (Supplementary Table S8). With regard to our nodal staging system, for example, we had to tradeoff between losing 15% of cases to gain the benefit of getting 37% more of controls in the development data set (42% and 52% in the validation data set).

When it comes to an overall ranking of the models, the pretreatment model showed the highest value of NRI (up to 53%) (Supplementary Table S8). The second ranked model was our nodal staging system due to having an advantage over OUTBACK eligibility criteria and EMBRACE criteria (up to 38%). The third and the fourth ranked models were EMBRACE criteria and OUTBACK eligibility criteria respectively. In addition, decision curve analysis of our pretreatment model and our new nodal staging system showed a moderate benefit for the treatment decisions for patients with the lower threshold at 12% and 15% respectively, compared to the higher threshold of EMBRACE criteria (18%) and OUTBACK eligibility criteria (24%) (Fig. 1C,D), confirming the impression of overall ranking of the model from NRI.

### Net reclassification in specific subgroups

#### Eligibility criteria of OUTBACK and high risk group of EMBRACE criteria

We performed additional NRI analysis in these specific subgroups of patients who are candidates for adjuvant chemotherapy, (Table 4). With 100 patients using the eligibility criteria of OUTBACK, the nodal staging system moved 3 patients to the very high-risk group. Even though this was a very small number, the very high-risk group was very specific for the development of distant metastases. Based on our analysis, overtreatment would occur in only 0.4 patients per 100 patients. In addition, 31 patients were moved to the high-risk group (overtreatment, 19 patients); 38 patients were moved to the low-risk group (undertreatment, 6 patients); but the groupings of 28 patients did not change. Similarly, our pretreatment model moved 29 patients to the high-risk group (overtreatment, 14 patients); 34 patients to the low-risk group (undertreatment, 3 patients); while 37 patients retained their original OUTBACK eligibility status. A more or less similar result was found for the validation data set. In short, our nodal system,the pretreatment and the posttreatment model safely moved patients to the low-risk group, thus avoiding unnecessary aggressive treatment in 84% to 91% of cases in the development data set and 70% to 84% of cases in the validation data set. On the other hand, to move to the high-risk group, we had some trade-off with overtreatment: 48% to 60% in the development data set, and 25% to 30% in the validation data set. Interestingly, if cases were moved to the minor lymphatic pathway (the very high-risk group) in the nodal staging system, this resulted in the highest probability of developing distant metastases (87%). The result even reached a 100% specificity with the validation data set.

Then we compared our models using patients at high risk according to the EMBRACE criteria (Table 4). With 100 patients, the nodal staging system moved 10 patients to the very high-risk group. Overtreatment would occur in 3 patients per 100 patients. The groupings of 90 patients did not change. Similarly, our pretreatment model 27 patients to the low-risk group (undertreatment, 6 patients); while 73 patients retained their original EMBRACE high risk group.

## Discussion

This study developed a clinical model for the prediction of distant metastases in locally advanced cervical cancer. Our pretreatment prediction model had only 2% or 3% less discrimination performance than the model of the Korean Gynecologic Oncology Group^7^. The use of FDG PET and squamous cell carcinoma antigen levels is costly and not available in 95% of middle-income countries^28^. However, we used computed tomography, which is routinely used around the world. We used simple predictors such as other lymph nodes, T stage, histology, initial hemoglobin, tumor size, and patient age. Even though our model was developed from these simple predictors, our discriminative performance (0.708) is adequate when compared with other studies. For example, a recent nomogram developed from lymph node parameters (site, number) and these clinical parameters resulted in a concordance index of 0.67 for predicting distant metastases^29^. In addition, our pretreatment prediction model has good clinical implications for reclassification. For example, our prediction model shifted approximately two-thirds of OUTBACK-eligible patients (one-third to high risk, and one-third to low risk). Among the low-risk cases, our model correctly classified more than 90% of these patients as not having distant metastases. Also, among the high-risk cases, our model correctly classified half of these patients as having distant metastases. In the same way, our prediction model shifted one-fourth of high-risk patients according to the EMBRACE criteria (all to intermediate or low risk). Among these lower-risk cases, our model correctly classified approximately 80% of these patients as not having distant metastases. This 10% difference could be explained by a difference of original categorization of OUTBACK eligibility criteria and EMBRACE criteria. OUTBACK eligibility criteria are very broad, ranging from FIGO (2009) stageIB1 with lymph node metastases to FIGO (2009) stage IVA. On the other hand, high risk group of EMBRACE criteria are very specific, presence of positive three or more nodes or positive nodes at common iliac or above.

One of the most important features of the nodal staging system is that it is integrated with very high-risk distant metastases in two or more positive minor lymphatic pathways (presacral or internal iliac lymph nodes). Our discriminative performance of this simple model (0.66) is compatible with sophisticated studies. For example, a comprehensive investigation of prediction models using the magnetic resonance imaging radiomics features of cervical masses and lymph nodes^30^ reported a concordance index of 0.66 in the development data set. In addition, the new nodal staging system proved to be the most important variable in our analysis using the Breiman mutation method. We also established that the nodal staging system could be used as an easy tool to aid decisions about whether to proceed to more aggressive treatment. The benefit is clearer with the OUTBACK or ACTLACC trial eligibility criteria. As well, in a comparison of the nodal staging system with the original EMBRACE, the criterion of the minor lymphatic pathway is very specific. If patients move to this category, the probability of distant metastases is very high. With very specific distant metastases in the minor lymphatic pathway, we propose that this pathway could be integrated as N3 and that our nodal staging could be the next nodal staging of the FIGO or AJCC staging system. In this study, we found that our nodal staging system still provided a net benefit of 10% to 20% in a tradeoff between losing cases and avoiding unnecessary controls. However, if patients are in the very high-risk group, the probability of metastasis is very specific and very high.

Our study has some limitations. First, adenosquamous carcinoma in our study was reported before the 2014 WHO standardized criteria^31^ and was not centrally reviewed. Also, hazard ratio of this type of histology was in the opposite direction with adenocarcinoma. Therefore, we combined this type of histology with squamous cell carcinoma. Secondly, consistent with the retrospective cohort nature of this research, some causes of death were unknown. As this might reflect missing distant metastases, there may have been an underestimation of risk. For example, even though there was good calibration in our development data, the calibration in our validation data set was mostly underestimated, as represented in our predicted curve above all the low-, intermediate-, and high-risk groups. Thirdly, our lymph nodes were not confirmed by PET/CT or pathology. However, we used the well-known criteria of positivity by size and high-risk features.

In summary, the new nodal staging system with the minor lymphatic pathway and the pretreatment model showed good standard model performance and good clinical implication of reclassification. These were also proven with external validation. This could differentiate between high-risk and low-risk patients, thus facilitating decisions to provide more aggressive treatment to prevent distant metastases.

## Supplementary Information

Supplementary Information.

## Data Availability

The data that support the findings of this study are available from the corresponding author, [JS], upon reasonable request.
