# Supplementary material for: Development and validation of a prognostic prediction model including the minor lymphatic pathway for distant metastases in cervical cancer patients
Source: Sci Rep. 2022 Jun 14;12:9873. doi: 10.1038/s41598-022-13616-0 (PMC9197836; doi:10.1038/s41598-022-13616-0)
Supplement: Supplementary file 1 — Supplementary Information. [file 41598_2022_13616_MOESM1_ESM.docx]

**Figure S1:** Flow of participants in development data set


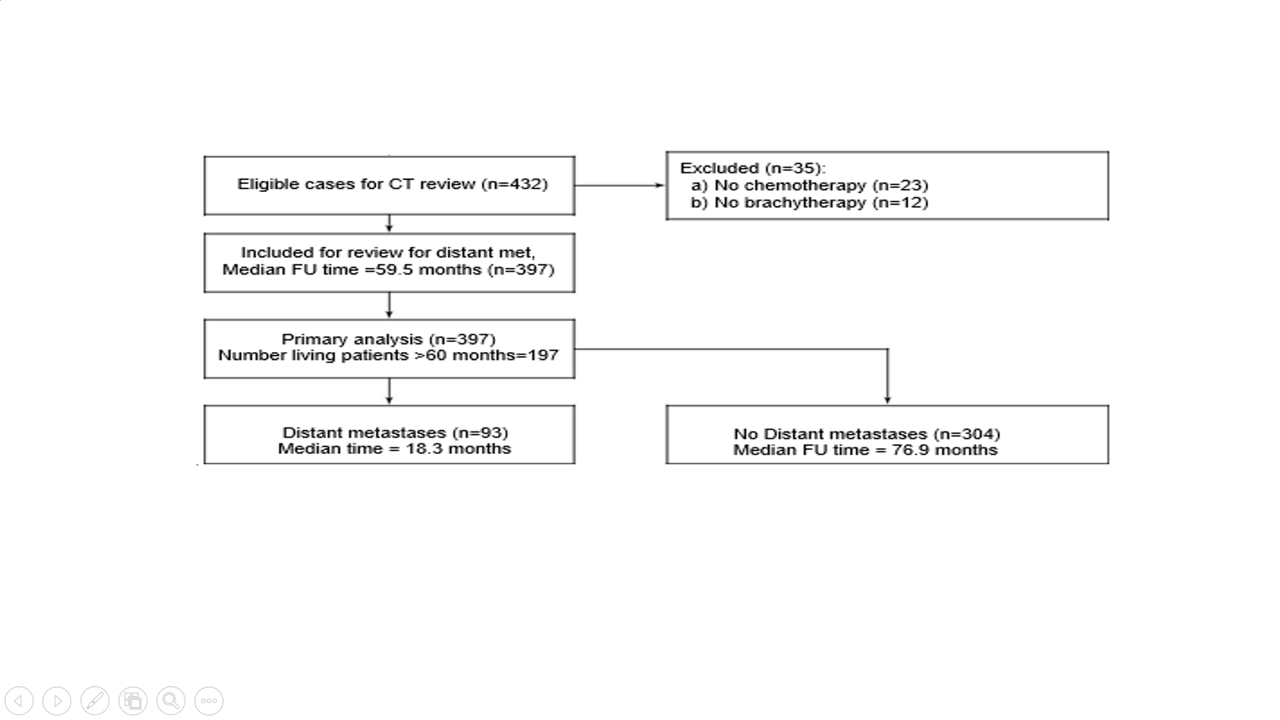


**Table S1:** Para-aortic lymphadenopathy: chain and level

| **Characteristics** | **All patients**  **(N=397)** | **Distant metastases**  **N(%)** | | **HR**  **(95%CI)** | ***P* value*** |
| --- | --- | --- | --- | --- | --- |
|  |  | **Yes=93** | **No=304** |  |  |
| **Chain of para-aortic LN** |  |  |  |  | <0.001 |
| Negative LN | 290(73.1) | 56(60.2) | 234(77.0) | 1 (reference) |  |
| Left aortic chain only | 49(12.3) | 13(14.0) | 36(11.8) | 1.44(0.79-2.63) |  |
| Aorto-caval only | 14(3.5) | 5(5.4) | 9(3.0) | 2.90(1.16-7.25) |  |
| Left aortic + Aorto-caval | 27(6.8) | 14(15.1) | 13(4.3) | 3.42(1.90-6.16) |  |
| Presence of Caval | 7(1.8) | 2(2.2) | 5(1.6) | 1.95(0.48-7.98) |  |
| All positive | 10(2.5) | 3(3.2) | 7(2.3) | 2.31(0.72-7.41) |  |
|  |  |  |  |  |  |
| **Highest level of**  **para-aortic LN**** |  |  |  |  | 0.001 |
|  |  |  |  |  |  |
| Negative LN | 290(73.1) | 56(60.2) | 234(77.0) | 1 (reference) |  |
| 326 b2 | 25(6.3) | 7(7.5) | 18(5.9) | 1.61(0.73-3.53) |  |
| 326 b1 | 61(15.4) | 21(22.6) | 40(13.2) | 2.07(1.25-3.42) |  |
| 326 a2 | 16(4.0) | 8(8.6) | 8(2.6) | 3.98(1.89-8.37) |  |
| 326 a1 | 5(1.3) | 1(1.1) | 4(1.3) | 1.45(0.20-10.48) |  |
|  |  |  |  |  |  |
|  |  |  |  |  |  |

**P* value of test parameters from univariable Cox regression

** According to Japan Society of Gynecologic Oncology

**Table S2:** Comparison of participant characteristics of the development and validation data sets

| **Characteristics** | **Development**  **(N=397)** | **External validation**  **(N=384)** | ***P* value** |
| --- | --- | --- | --- |
| **Demographic & tumor** |  |  |  |
| Age, mean ± SD | 55.1(11.8) | 52.6(11.0) | 0.003 |
| Initial hemoglobin (g/dl)* | 11.4 (1.8) | 11.5 (1.7) | 0.61 |
| Histology, N(%)** |  |  | 0.150 |
| SCC + AdenoSCC | 327(82.4) | 300(78.1) |  |
| AdenoCA | 70(17.6) | 84(22.0) |  |
| Tumor size, mean ± SD** | 4.4(1.5) | 4.3(1.5) | 0.430 |
| **Staging** |  |  |  |
| FIGO 2018, N(%) |  |  | <0.001 |
| I–II | 67(16.9) | 116(30.2) |  |
| IIIB | 132(33.2) | 103(26.8) |  |
| IIIC1 | 124(31.2) | 121(31.5) |  |
| IIIC2 | 70(17.6) | 32(8.3) |  |
| IVA | 4(1.0) | 12(3.1) |  |
| T stage only |  |  | <0.001 |
| I–II | 200 (50.4) | 239 (62.2) |  |
| III–IV | 197 (49.6) | 145 (37.8) |  |
| OUTBACK |  |  | <0.001 |
| < Eligibility criteria | 5 (1.3) | 8(2.1) |  |
| Eligibility criteria | 322(81.1) | 344(89.6) |  |
| > Eligibility criteria | 70(17.6) | 32(8.3) |  |
| Original EMBRACE |  |  | <0.001 |
| Low risk | 9 (2.3%) | 20 (5.2%) |  |
| Intermediate risk | 212 (53.4%) | 256 (66.7%) |  |
| High risk | 176 (44.3%) | 108 (28.1%) |  |
| Our nodal staging system |  |  | <0.001 |
| Low risk | 134 (33.8%) | 211 (54.9%) |  |
| Intermediate risk | 86 (21.7%) | 65 (16.9%) |  |
| High risk | 160 (40.3%) | 104 (27.1%) |  |
| Minor lymphatic pathway | 17 (4.3%) | 4 (1.0%) |  |
| **Treatment** |  |  |  |
| Concurrent chemo-RT, N(%) |  |  | <0.001 |
| Weekly | 381(96.0) | 239(62.2) |  |
| Monthly | 16(4.0) | 145(37.8) |  |
| Treatment time |  |  | <0.001 |
| ≤ 55 days | 310 (78.1%) | 213 (55.5%) |  |
| > 55 days | 87 (21.9%) | 171 (44.5%) |  |
| Response at 1 month |  |  | 0.200 |
| Response | 372 (93.7%) | 368 (95.8%) |  |
| No response | 25 (6.3%) | 16 (4.2%) |  |
| **Outcome** |  |  |  |
| 1^st^ distant metastases, N(%) | 93(23.4) | 76(19.8) | 0.220 |
| 1^st^ local failure, N(%) | 67(16.9) | 45(11.7) | 0.042 |
| 1^st^ regional failure, N(%) | 22(5.5) | 29(7.6) | 0.310 |
| Death, N(%) | 176(44.3) | 124(32.3) | <0.001 |
|  |  |  |  |

12 individuals have 1 missing value; 1 individual has 2 missing values (tumor size and histology)

*Missing data =10 in validation set; **missing data = 2 in validation set. No clear outcome-related reasons for any missing data

**Figure S2:** Comparison of linear predictor in development and validation data sets in both pretreatment and posttreatment model


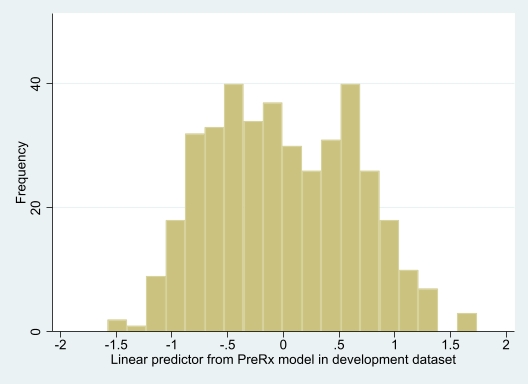

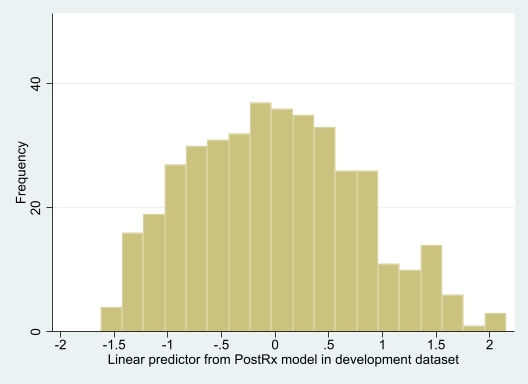


Mean 8.05e-09, sd .7608529, median -.006927 (25% -.6042877 to 75% .5865921) Mean -3.42e-09, sd .7902118, median -.0289859 (25%-.6085236 to 75% .5443001)


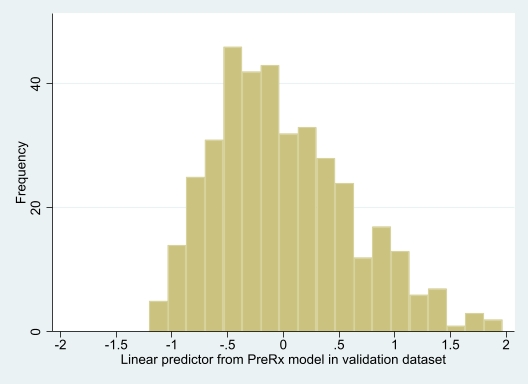

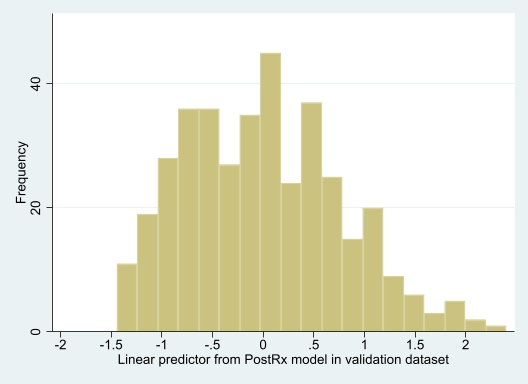


Mean -4.92e-09, sd .7543226, median -.0647406 (25% -.6542041to 75% .4386781) Mean 7.04e-09, sd .773977, median -.0349312 (25% -.6258194 to 75% .5187538)

**Table S3:** Backward elimination of subset and forcing of clinical factors back into the model

| Model | LR test of model | *P* value |
| --- | --- | --- |
| **Lymph node system** |  |  |
| 1. New nodal system + PAN* chain + PAN level |  |  |
| 2. New nodal system + PAN level | 2 vs 1 | 0.2636 |
| 3. New nodal system | 3 vs 1 | 0.4012 |
|  |  |  |
| **Add clinical stage and tumor size** |  |  |
| 4. New nodal system + clinical T stage + tumor size | 4 vs 3 | 0.0318 |
| 5. New nodal system + clinical T stage | 5 vs 4 | 0.3437 |
|  |  |  |
| **Add histology** |  |  |
| 6. New nodal system + clinical T stage + histology | 6 vs 5 | 0.0104 |
|  |  |  |
| **Add Hemoglobin and age** |  |  |
| 7. New nodal system + clinical T stage + histology + initial Hb** + age | 7 vs 6 | 0.1510 |
| 8. New nodal system + clinical T stage + histology + initial Hb | 8 vs 7 | 0.8767 |
|  |  |  |
| **Force tumor size and age back** |  |  |
| 9. New nodal system + clinical T stage + histology + initial Hb + tumor size + age | 9 vs 8 | 0.2765 |

* PAN, Para-aortic lymph nodes; **Hb, Hemoglobin

**Table S4:** model specification of ACTLACC and the post-treatment model

| **ACTLACC**  (grouping) | **Low risk** (Less stage): stage IB1(FIGO 2009)  **Intermediate risk** (eligibility of trial): stage IIB to IVA (FIGO 2009)  **High risk** (More stage): positive para-aortic lymph node |
| --- | --- |
| **Model after treatment**  (probability prediction then grouping by 15%, 30% risk) | Baseline survival at 60 months (S0_60m) = .9056749  Shrinkage factor = 0.8361  Linear predictor (LP) = .5274445* intermediate@our nodal system + 1.040608* high@our nodal system + 1.731063* minor@our nodal system + .4254939*clinicalT34 + .7551457*adeno + -.1118243*( initialHb-11.44861461) + .0809189* (tumorsize-4.412989926) + .0148026*(Age-55.07808564) + .4104035*treatmenttimemore55days(>55days =1) + .2666545* TreatmentResponse1mo(no response=1)  5-year risk of distant metastasis = 1- (S0_60m^ exp(LP*0.8361))  **Low risk**: < 15% of distant metastasis  **Intermediate risk**: 15-30% of distant metastasis  **High risk**: > 30% of distant metastasis |

**Table S5:** Discrimination performance of additional models in development and validation data sets

|  | **Standard** | | **Full model:** |
| --- | --- | --- | --- |
|  | **Eligibility:**  **ACTLACC** | | **Prognostic model**  **after treatment** |
| **DEVELOPMENT** |  | |  |
| N | 397 | | 397 |
| Number of events | 93 | | 93 |
|  |  | |  |
| **Variables** |  | |  |
| Trial eligibility |  | |  |
| Stage under | 1 (reference) | | - |
| Eligible | 0.59(0.15-2.43) | | - |
| Stage over | 1.38(0.33-5.82) | | - |
|  |  | |  |
| EMBRACE original |  | |  |
| Low risk | - | |  |
| Intermediate risk | - | | - |
| high risk | - | | - |
|  |  | | - |
| Our nodal system |  | |  |
| Low risk | - | | 1 (reference) |
| Intermediate risk | - | | 1.69(0.84-3.40) |
| High risk | - | | 2.83(1.59-5.05) |
| Minor pathway | - | | 5.65(2.46-12.97) |
|  |  | |  |
| T stage only |  | |  |
| I–II | - | | 1 (reference) |
| III–IV | - | | 1.53(0.97-2.41) |
|  |  | |  |
| Histology |  | |  |
| SCC + adenoSCC | - | | 1 (reference) |
| AdenoCA | - | | 2.13(1.26-3.58) |
|  |  | |  |
| Initial Hb | - | | 0.89(0.79-1.01) |
| Tumor size | - | | 1.08(0.93-1.26) |
| Age | - | | 1.01(0.996-1.03) |
| Treat. time > 55 days | - | | 1.51(0.95-2.38) |
| No Response at1 mo. | - | | 1.31(0.54-3.18) |
|  |  | |  |
| Cox 2-yr DM(%) | 14.48 | | 12.35 |
| Cox 3-yr DM(%) | 20.17 | | 17.79 |
| Cox 5-yr DM(%) | 27.05 | | 24.84 |
| C-statistics(95%CI) | 0.574(0.527-0.621) | | 0.716(0.665-0.767) |
| 1-Optimism | 0.9311 | | 0.8361 |
| Optimism corrected | 0.571(0.524-0.618) | | 0.687(0.640-0.735) |
|  |  | |  |
| **VALIDATION** |  | |  |
| N | 384 | | 384 |
| Number of events | 76 | | 76 |
| C-statistics(95%CI) | 0.522(0.480-0.564) | | 0.718(0.665-0.771) |
|  |  | |  |
|  |  |  |  |

ACTLACC stage under = IA2-IB1, eligible = IIB to IVA, stage over = PAN+

*DM= distant metastasis rate

**Figure S3:** Calibration performance of additional model in development and validation data sets


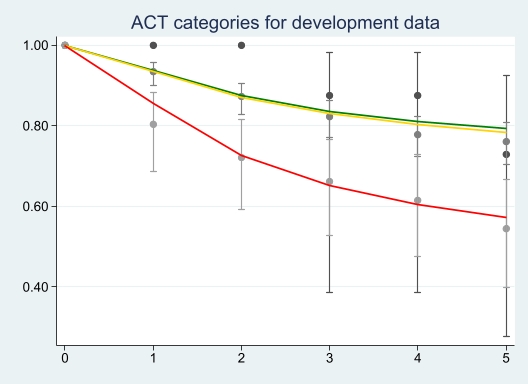

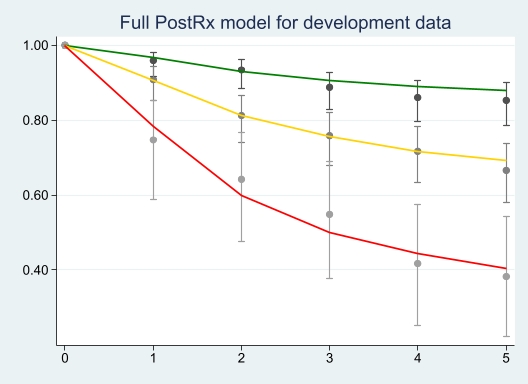


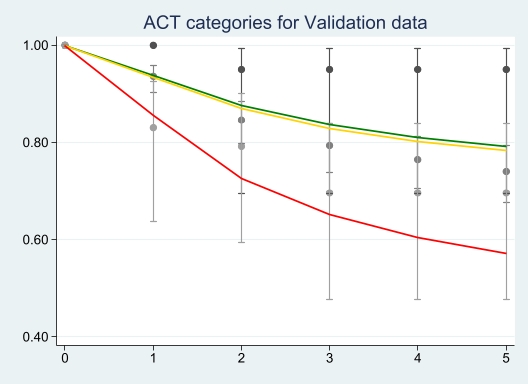

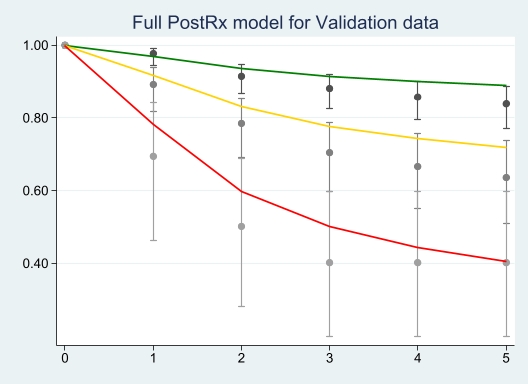


**Figure S4**: Diagonal line calibration curve at time point 5 year in development and validation data sets

Development: Pretreatment model Development: Posttreatment model


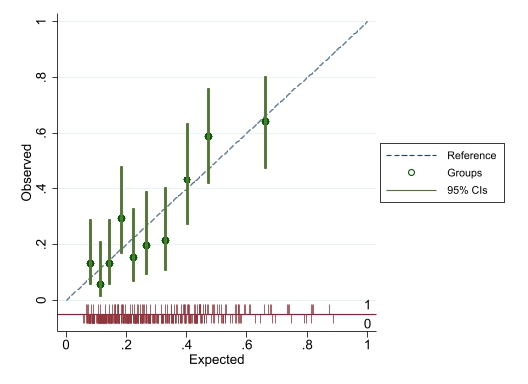

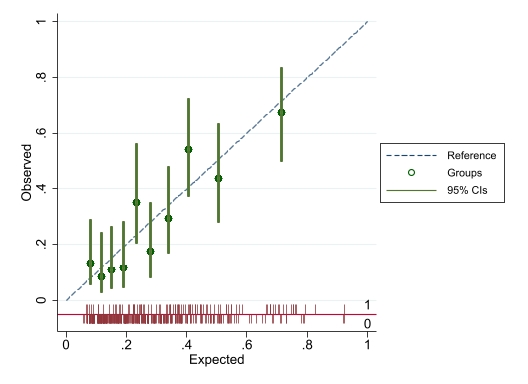


Validation: Pretreatment model Validation Posttreatment model


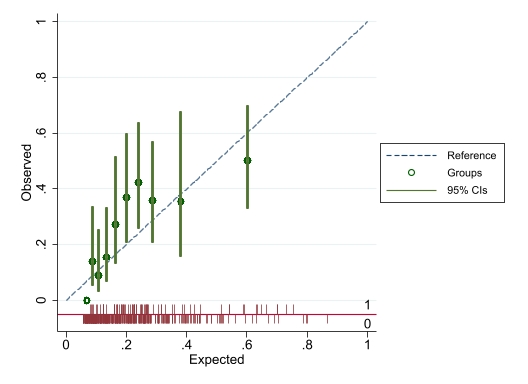

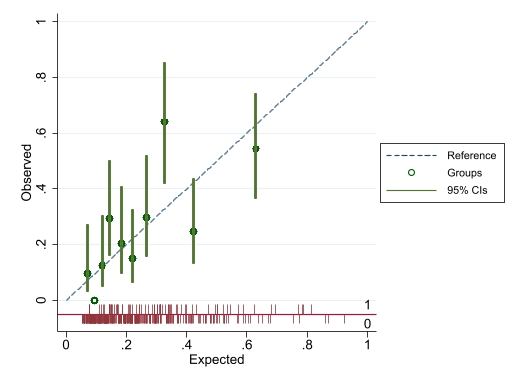


**Table S6:** Quantitative method of calibration performance: hazard ratios of the risk groups and *P* value of mis-specification

|  | OUTBACK | ACTLACC | EMBRACE to level A1 | Our nodal system | Pretreatment model | Posttreatment model |
| --- | --- | --- | --- | --- | --- | --- |
| **DEVELOPMENT** |  |  |  |  |  |  |
| HR group 2 vs 1 | 0.59(0.43) | 1.05(0.75) | 0.79(0.57) | 1.78(0.62) | 2.86(1.11) | 2.57(0.95) |
| HR group 3 vs 1 | 1.38(1.01) | 2.40(1.76) | 2.18(1.57) | 3.66(1.03) | 9.19(3.53) | 8.02(2.94) |
|  |  |  |  |  |  |  |
| **VALIDATION** |  |  |  |  |  |  |
| Calibration slope | - | - | - | - | 0.868 | 0.893 |
| Mis-specification p value | - | - | - | - | 0.586 | 0.358 |
| HR group 2 vs 1 | 1.71(1.73) | 6.20(6.26) | 4.95(5.01) | 1.40(0.47) | 3.71(1.53) | 3.81(1.69) |
| HR group 3 vs 1 | 2.89(3.05) | 9.78(10.32) | 10.36(10.54) | 2.46(0.62) | 6.42(2.70) | 9.05(4.00) |

* Original categorized

** Probability prediction and grouping to 3 groups at 25, 75 percentiles of linear predictor

**Table S7:** Model performance after removing patients treated with monthly chemotherapy and prophylactic paraaortic radiotherapy.

|  | **OUTBACK** | **Original** | | **Simplified model:** | | **Full model:** | |
| --- | --- | --- | --- | --- | --- | --- | --- |
|  | **Eligibility**  **criteria** | **EMBRACE**  **(to level A1*)** | | **New nodal**  **staging system** | | **Prognostic model**  **before treatment** | |
| **DEVELOPMENT** |  |  | |  | |  | |
| Number of patients | 361 | 361 | | 361 | | 361 | |
| Number of events | 75 | 75 | | 75 | | 75 | |
|  |  |  | |  | |  | |
| C-statistics(95%CI) | 0.546(0.498-0.594) | 0.627(0.570-0.682) | | 0.656(0.601-0.712) | | 0.715(0.656-0.774) | |
| 1-Optimism | 0.9850 | 0.9586 | | 0.9446 | | 0.8484 | |
| Optimism corrected | 0.538(0.494-0.582) | 0.622(0.567-0.677) | | 0.651(0.592-0.709) | | 0.688(0.632-0.743) | |
|  |  |  | |  | |  | |
| **VALIDATION** |  |  | |  | |  | |
| Number of patients | 231 | 231 | | 231 | | 231 | |
| Number of events | 37 | 37 | | 37 | | 37 | |
|  |  |  | |  | |  | |
| C-statistics(95%CI) | 0.497(0.446-0.549) | 0.633(0.547-0.719) | | 0.675(0.593-0.756) | | 0.717(0.643-0.792) | |
|  |  |  | |  | |  | |
|  |  | |  | |  | |  |

* EMBRACE extended to level A1 (node level just below diaphragm) in order to fairly compare with other models

**OUTBACK: Less than eligibility criteria = IA2-IB1, eligibility criteria = IB1N+ to IVA, more than eligibility criteria = PAN+

***DM= distant metastasis rate

**Figure S5**: Calibration curves after removing patients treated with monthly chemotherapy and prophylactic paraaortic radiotherapy


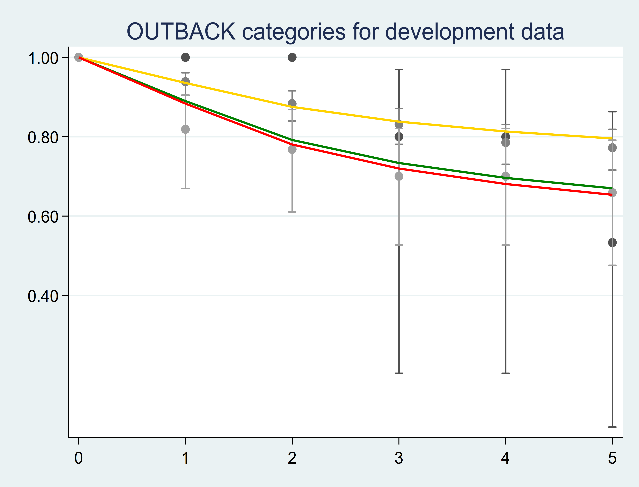

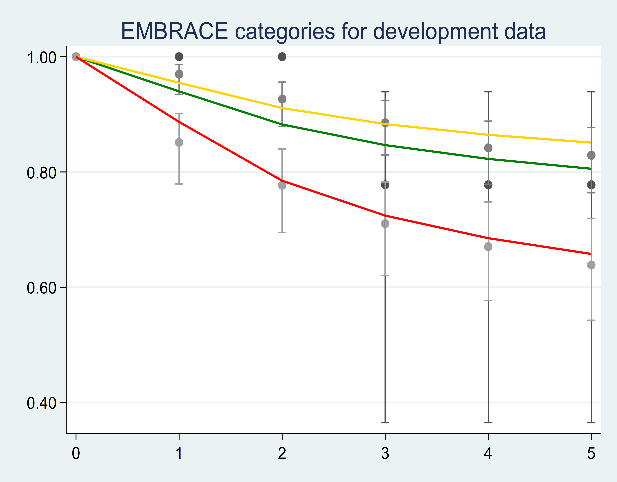


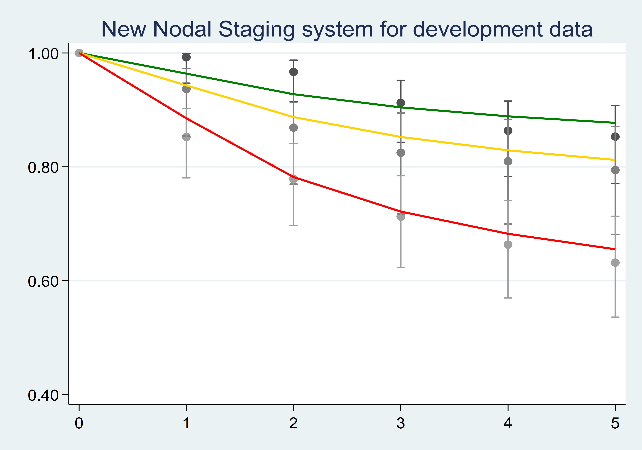

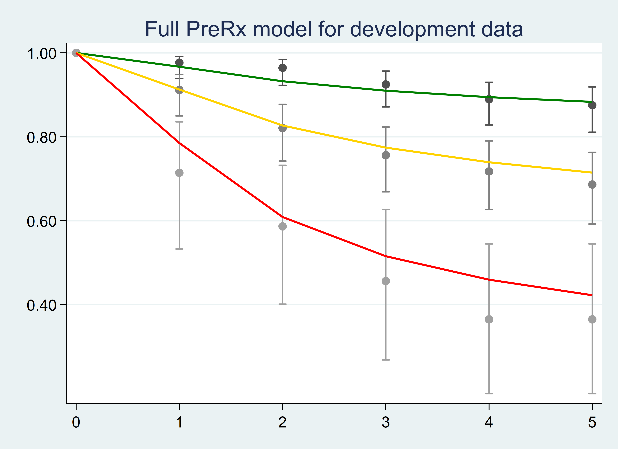


**Table S8:** Net reclassification improvement (NRI) at 5 years with 100 bootstrapping between standard and new grouping/model in development and validation data sets

|  | Nodal staging system  % (range from 100 bootstrapping) | | | Pre-treatment model  % (range from 100 bootstrapping) | | |
| --- | --- | --- | --- | --- | --- | --- |
|  | NRI+ | NRI- | NRI | NRI+ | NRI- | NRI |
| Development |  |  |  |  |  |  |
| OUTBACK | 22  (9 to 35) | 16  (7 to 28) | 38  (20 to 53) | 31  (18 to 45) | 18  (8 to 28) | 48  (30 to 63) |
|  |  |  |  |  |  |  |
| Embrace to A1 level | -15  (-24 to 7) | 37  (32 to 44) | 22  (13-35) | -6  (-16 to 3) | 39  (32 to 46) | 32  (20 to 45) |
| Validation |  |  |  |  |  |  |
| OUTBACK | -11  (-34 to 9) | 40  (31 to 48) | 29  (4 to 51) | 12  (-3 to 28) | 41  (32 to 48) | 53  (35 to 72) |
|  |  |  |  |  |  |  |
| Embrace to A1 level | -42  (-51 to 32) | 52  (47 to 58) | 10  (-0.1 to 23) | -19  (-33 to -2) | 53  (46 to 59) | 34  (14 to52) |

NRI+ (net reclassification in patients with distant metastasis)

NRI- (net reclassification in patients without distant metastasis)

NRI= summation of NRI+ and NRI-
